# The burden of gastrointestinal diseases in Japan, 1990–2019, and projections for 2035

**DOI:** 10.1002/jgh3.12883

**Published:** 2023-03-07

**Authors:** Yasutoshi Shiratori, Susan Hutfless, George Rateb, Katsuyuki Fukuda

**Affiliations:** ^1^ Department of Gastroenterology St. Luke's International Hospital Tokyo Japan; ^2^ Department of Gastroenterology Sherbrooke University Hospital Quebec Canada; ^3^ Departments of Epidemiology and Gastroenterology Johns Hopkins University Baltimore Maryland USA

**Keywords:** aging society, cancers, gastrointestinal diseases, global burden of disease, health care

## Abstract

**Background and Aim:**

Disease burden estimation allows clinicians and policymakers to plan for future healthcare needs. Although advances have been made in gastroenterology, as Japan has an aging population, disease burden assessment is important. We aimed to report gastrointestinal disease burden in Japan since 1990 and project changes through to 2035.

**Methods:**

This descriptive study examined the crude and age‐standardized rates of prevalence, mortality, and disability‐adjusted life years (DALYs) of 22 gastrointestinal diseases between 1990 and 2019. We used data from the Global Burden of Disease study 2019. We calculated the expected disease burden of gastrointestinal diseases by 2035 using an autoregressive integrated moving average.

**Results:**

Since 1990, cancer has accounted for most gastrointestinal disease‐related causes of mortality and DALYs in Japan (77.1% and 71.2% in 1990, 79.2% and 73.7% in 2019, respectively). Although cancer‐associated age‐standardized mortality rates and DALYs have shown a decreasing trend, the crude rates have increased, suggesting that an aging society has a significant impact on the disease burden in Japan. Therefore, the overall gastrointestinal disease burden is expected to increase by 2035. Noncancerous chronic diseases with a high burden included cirrhosis, biliary disease, ileus, gastroesophageal reflux disorder, hernia, inflammatory bowel disease, enteric infections, and vascular intestinal disorders. In cirrhosis, the DALYs for hepatitis C decreased and the prevalence of non‐alcoholic steatohepatitis increased.

**Conclusion:**

In the super‐aging Japanese society, the burden of gastrointestinal diseases is expected to increase in the coming years. Colorectal, gastric, pancreatic, and liver cancers are the focus of early detection and treatment.

## Graphical abstract

Disease burden estimation allows clinicians and policymakers to plan for future healthcare needs. Our study provides comprehensive estimates of the burden of gastrointestinal diseases in a super‐aging society focusing on cancers and other chronic gastrointestinal diseases. Currently, colorectal, gastric, pancreatic, and liver cancers are the focus of early detection and treatment.

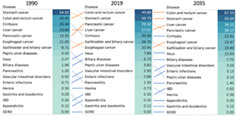

## Introduction

Gastrointestinal diseases include a wide variety of diseases, and their management depends on following a long‐term strategy based on the age structure of the population. Japan is the leading country in terms of an aging population. In 2020, 36.2 million people of the Japanese population were ≥65 years old, constituting 28.8% of the total population.^1^, ^2^ This percentage is extraordinarily high compared with the global figure of 9.2%, and is expected to further increase to 35% by the year 2040.^2^, ^3^ Because older people living with multiple morbidities require long‐term health care, the value of preventing and controlling chronic diseases has been emphasized by the World Health Organization (WHO).^4^

The Global Burden of Disease (GBD) study 2019 presents a comprehensive assessment of a variety of diseases in terms of prevalence, mortality, and disability‐adjusted life years (DALYs).^3^, ^5^ DALYs measure the overall disease burden including years lost due to morbidity, disability, and premature death. In other words, DALYs are defined as the sum of years of life lost (YLL) and years lived with a disability (YLD). It is an extension of the concept of potential years lost due to impaired health and disability; it has gained wide acceptance in recent years.^6^, ^7^, ^8^, ^9^, ^10^, ^11^, ^12^, ^13^, ^14^, ^15^ Since 1990, cancer has been the leading cause of mortality and DALYs in Japan.

To date, there has been no systematic evaluation of the burden of gastrointestinal diseases in Japan. Considering the changes associated with healthcare systems due to an accelerated aging society, and the availability of treatment options such as medication for *Helicobacter pylori* eradication and hepatitis C antiviral agents, it is important to know the burden of diseases to determine the priorities of medical resources and give future directions for research development.

Our study aimed to analyze the results of the GBD study in relation to gastrointestinal diseases between 1990 and 2019 in Japan, and predict the transition through to 2035. This study may guide the mapping of the burden of gastrointestinal diseases and contribute to formulating national health policies.

## Methods

### Overview

In this study, we included GBD 2019 data provided by the Institute for Health Metrics and Evaluation (IHME) to analyze the status of gastrointestinal diseases in Japan between 1990 and 2019. The GBD study results were compiled from 195 countries, based on 354 causes and 3484 sequelae. The data were obtained from 68 781 sources, including hospital and clinical data, inpatient and outpatient medical records, surveillance data, and other extensive literature.^7^, ^8^, ^9^, ^10^, ^11^, ^12^ Although age‐standardized data are useful for evaluating treatment progress, crude data are more reliable for evaluating real‐world scenarios and investments in medical resources. Therefore, we treated the crude data as primary data, which better reflected the changes in the burden of disease due to an aging society. Institutional Review Board approval was not required because this was a secondary analysis of publicly available GBD data.^16^

### Gastrointestinal diseases of interest

The prevalence rates, mortality, and DALYs of cancers, noncancerous chronic diseases, and digestive infections that accounted for the disease burden were examined. DALYs are calculated from the sum of YLD and YLL, and YLD is calculated as the product of the disease incidence, disease weight, and the number of years until improvement or death.^5^ Diseases were defined according to the International Classification of Diseases, 10th Revision. The cancers that were included in the study were esophageal, stomach, colorectal, liver, biliary tract, and pancreatic cancers. Noncancerous chronic diseases included cirrhosis and other chronic liver diseases, gastroesophageal reflux disorders (GERD), peptic ulcer disease, gastritis and duodenitis, appendicitis, ileus, hernias, inflammatory bowel disease (IBD), vascular bowel disease, gallbladder and biliary diseases, pancreatitis, and digestive infections. Digestive infections included enteric infections of bacterial, viral, and parasitic etiologies.

### Outcomes of interest

Crude and age‐standardized rates of prevalence, mortality, and DALYs per 10 000 were estimated, and these were the main outcomes of our study. Age standardization was performed using World's age criteria developed in line with the WHO age criteria.^4^

### Statistics

An autoregressive integrated moving average (ARIMA) model was used to forecast the burden of gastrointestinal diseases from 2020 to 2035.^17^, ^18^ Three main component parameters known as “P,” “D,” and “Q” determine the ARIMA. Autoregression is denoted by “P” and is the lag order; “D” stands for integrated and is the degree of differentiation; and “Q” signifies the moving average, specifically the order of the moving average.

Data were analyzed using Stata version 16 (Stata Corp., TX, USA) and Tableau 2021 (Tableau Software, WA, USA).

## Results

### Burden of cancers

**Figure 1:**
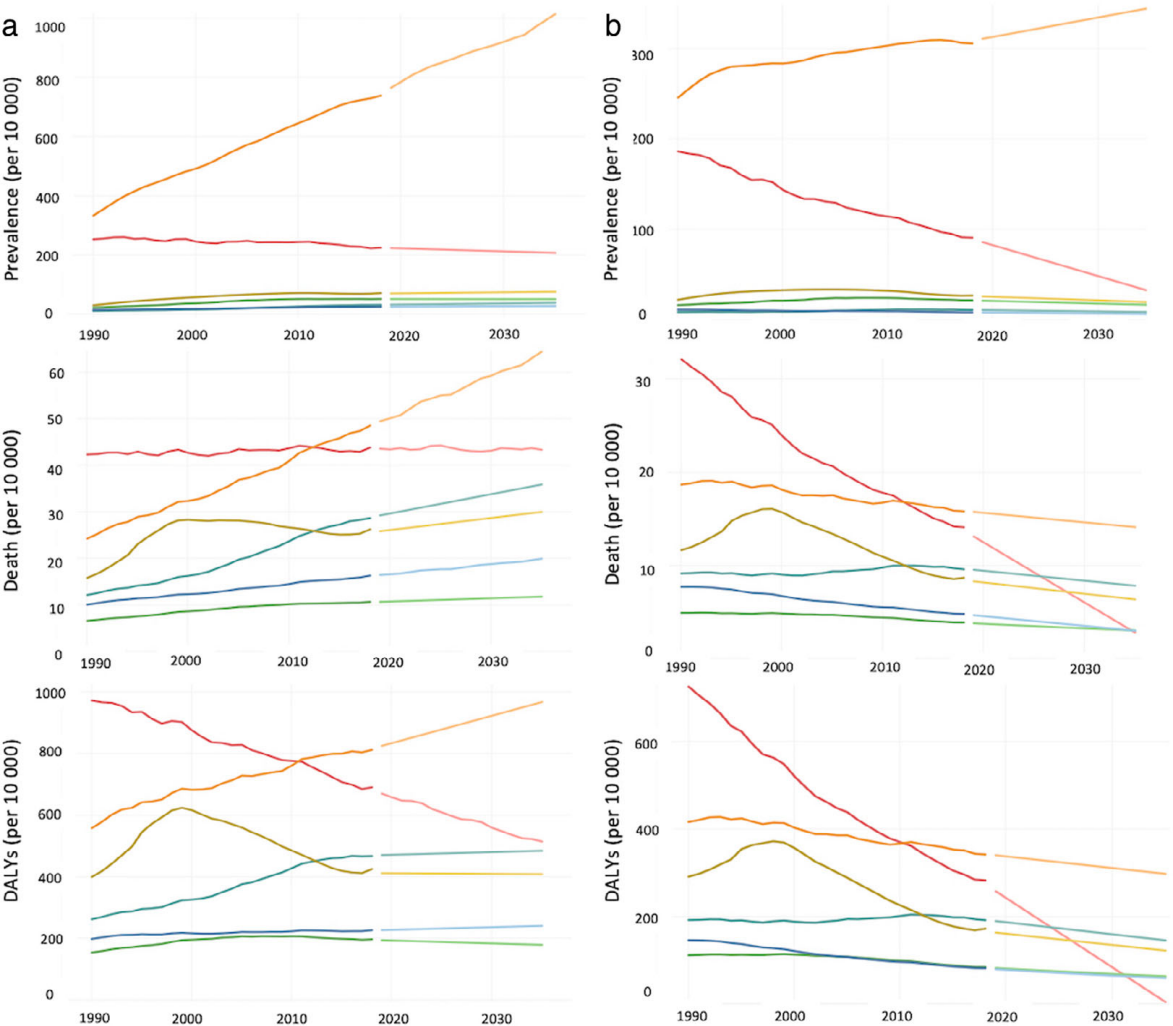
(a) Crude and (b) age‐standardized rates of prevalence, death, and disability‐adjusted life years (per 10 000) of gastrointestinal cancers in Japan, 1990–2035. 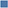, Biliary cancer, actual; 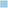, biliary cancer, estimate; 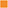, colorectal cancer, actual; 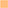, colorectal cancer, estimate; 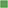, esophageal cancer, actual; 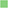, esophageal cancer, estimate; 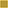, liver cancer, actual; 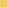, liver cancer, estimate; 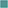, pancreas cancer, actual; 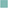, pancreas cancer, estimate; 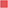, stomach cancer, actual; 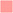, stomach cancer, estimate.

In 2019, cancer‐related deaths and DALYs accounted for 79.2% and 73.7%, respectively, of the disease burdens; these values were higher than those reported in 1990 (77.1% deaths and 71.2% DALYs, respectively). Moreover, it was estimated that these values will remain high (78.2% deaths and 73.2% DALYs) in the year 2035. Figure 1a shows the crude rates of prevalence, deaths, and DALYs related to cancer (per 10 000). The prevalence of colorectal cancer showed a steep rise until 2035. The top three cancer‐related death rates reported in 2019 were 49.9, 44.7, and 29.3 for colorectal, stomach, and pancreatic cancers, respectively. In 2035, the death rates were estimated to be 67.5, 56.6, and 38.5 for colorectal, stomach, and liver cancers, respectively. Likewise, DALYs in 2035 appeared high as 968.7, 515.6, and 485.6 for colorectal, stomach, and pancreatic cancers, respectively. In contrast to the results of crude analyses, age‐standardized rates of death and DALYs decreased, as shown in Figure 1b. In particular, the age‐standardized rates of prevalence, death, and DALYs related to stomach cancer showed a marked decrease.

### Burden of cirrhosis

**Figure 2:**
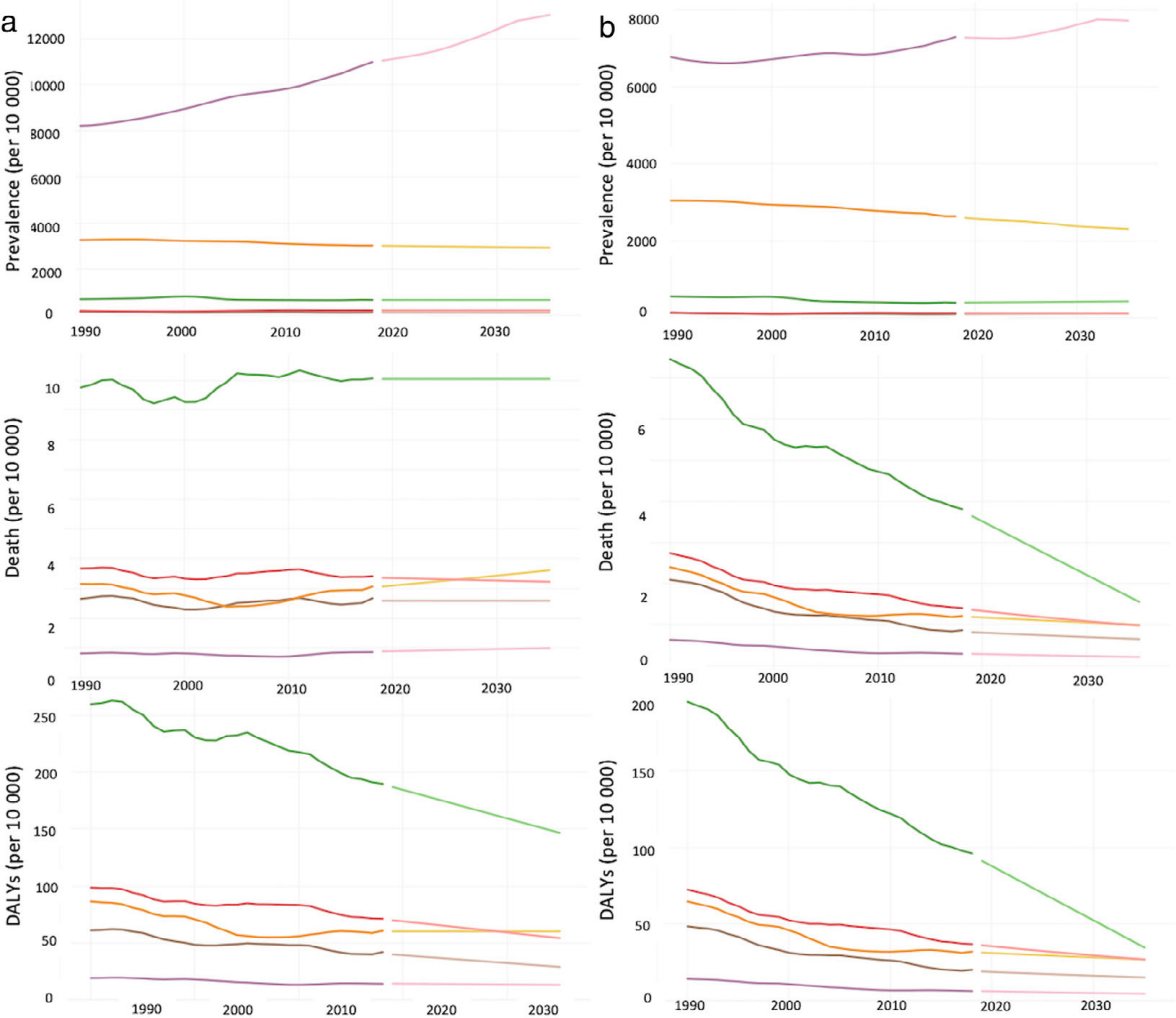
(a) Crude and (b) age‐standardized rates of prevalence, death, and disability‐adjusted life years (per 10 000) of cirrhosis in Japan, 1990–2035. 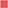, Alcohol cirrhosis, actual; 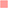, alcohol cirrhosis, estimate; 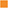, cirrhosis type B, actual; 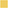, cirrhosis type B, estimate; 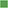, cirrhosis type C, actual; 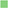, cirrhosis type C, estimate; 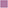, non‐alcoholic steatohepatitis (NASH), actual; 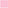, NASH, estimate; 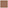, other cirrhosis, actual; 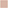, other cirrhosis, estimate.

In 2019, 9.1% of the total deaths and 9.9% of DALYs were associated with cirrhosis, which was lower than that reported in 1990 (13.9% of deaths and 14.8% of DALYs). Further, the death rate and DALYs were observed to decrease to 7.8% and 8.0%, respectively, by 2035. Figure 2a,b shows the tendency of crude and age‐standardized rates of prevalence, deaths, and DALYs for each cause of cirrhosis (per 10 000). The crude prevalence of non‐alcoholic steatohepatitis (NASH) markedly increased from 8237 in 1990 to 11 067 in 2019 and was estimated to be 13 053 by 2035. The crude rate of DALYs in patients with cirrhosis due to hepatitis C decreased from 260.3 in 1990 to 187.2 in 2019 and was estimated to be 147.2 by 2035.

### Changes in the burden of total gastrointestinal diseases

**Figure 3:**
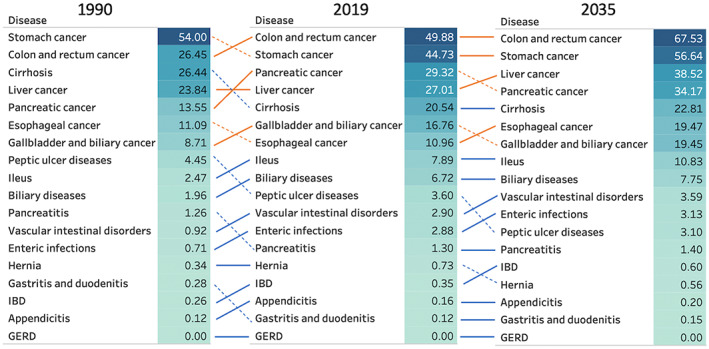
Changes in death rates (per 10 000) for both sexes combined in 1990, 2019, and 2035.

**Figure 4:**
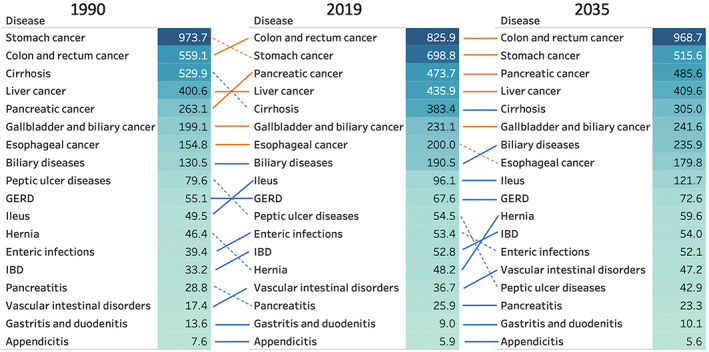
Changes in disability‐adjusted life years (per 10 000) for both sexes combined in 1990, 2019, and 2035.

Figures 3 and 4 show the changes in death and DALY rates associated with gastrointestinal diseases, respectively. The total number of deaths (per 10 000) was 144.7 in 1990 and 225.8 in 2019; it was estimated to be 262.7 by 2035. Similarly, the total number of DALYs (per 10 000) was 3581.6 in 1990 and 3889.5 in 2019 and was estimated to be 3830.9 by 2035. The top three causes of death were the following: in 1990, stomach cancer, colorectal cancer, and cirrhosis; in 2019, stomach cancer, colorectal cancer, and liver cancer; and in 2035, likely to be colorectal cancer, stomach cancer, and pancreatic cancer. Among men, colorectal and stomach cancers remained the two leading causes of death from 2019 to 2035 (Figure S1, Supporting information). Among women, colorectal and stomach cancers were the top two causes of death in 2019, but pancreatic cancer was observed to be the second highest cause of death in 2035 following colorectal cancer (Figure S2). Among noncancerous chronic diseases, the death rate of cirrhosis was the highest, followed by ileus, biliary disease, vascular bowel disease, enteral infections, and peptic ulcer disease (Fig. 3). For DALYs, cirrhosis was the highest, followed by biliary disease, ileus, GERD, hernia, and IBD (Fig. 4).

## Discussion

We analyzed the burden of gastrointestinal diseases in Japan using data from the GBD 2019 study,^5^ which is supported and recognized as a standard for international epidemiologic comparisons by the WHO. The GBD study has been accepted as a worldwide epidemiological study because the data are extremely useful for international comparisons and healthy life expectancy is estimated to be the same, objectively, worldwide over a long period of time.^5^, ^7^, ^8^, ^9^, ^10^, ^11^, ^12^ Additionally, there was no significant discrepancy between the GBD data and Japanese vital registration by the Ministry of Health, Labour and Welfare.^14^, ^15^ We used prevalence, death, and DALYs as outcomes. Prevalence is affected by the incidence, duration of disease (prognosis and development of treatment), and population migration. Colorectal cancer has a relatively good prognosis in addition to the development of treatment. In contrast, pancreatic cancer has a poor prognosis and tends to have a low prevalence.

Our study used crude data as the primary data and age‐standardized data to make comparisons. As age‐standardized data were useful for assessing treatment progress, we hypothesized that crude data would be more reliable for assessing the real‐life scenario of an aging society and investment in healthcare resources. Age‐standardized rates of mortality and DALYs showed improvements because of screening and delivery of appropriate treatments; however, the aging society affects the burden of disease, and the burden of diseases,^2^, ^3^ mainly of cancer, is expected to increase in future.^19^, ^20^ Our study provides information on the future trend of the burden of gastrointestinal diseases, and both policymakers and physicians need to be aware of this trend.

Cancer is the leading cause of death in Japan, and the burden of cancer will continue to increase for at least the next few decades.^21^ Among specific gastrointestinal cancers, colorectal cancer was a major contributor to the disease burden in 2019, followed by cancers of the stomach, pancreas, liver, biliary duct, and esophagus. According to the National Cancer Center data, colorectal and gastric cancers are the second and third leading causes of cancer death respectively after lung cancer.^22^ Measures such as fecal occult blood screening, early‐stage disease detection by colonoscopy, and the development of surgical techniques or chemotherapy regimens may contribute to decreasing the deaths due to colorectal cancer^7^; however, the burden of colorectal cancer will continue to increase in this aging society. A substantial decrease in age‐standardized death rates and DALYs of stomach cancer may be associated with *H. pylori* eradication, screening programs, and decreased smoking prevalence.^15^ A previous descriptive study revealed that sex differences in the incidence of gastric cancer were higher among older patients.^13^ These findings highlight the emphasis of having a sex‐sensitive health policy to cope with the burden of cancers. If *H. pylori* infection rates decline, future screening intervals should be reconsidered from the perspective of cost‐effectiveness.^11^, ^15^ Pancreatic cancer is typically diagnosed in older people, and it has been associated with a high risk of death.^8^ Further, pancreatic cancer is usually diagnosed at an advanced stage and has a poor response to chemotherapy. Ideally, population‐level screening modalities would be necessary to enhance early detection. The burden of liver cancer remains high despite treatment for hepatitis C virus and a decline in alcohol consumption.^23^ Further, changes have been observed in the etiology of cirrhosis, with NASH currently showing the highest prevalence rate.^24^ The burden of esophageal cancer is decreasing due to the decline in smoking rates and the effect of gastroscopic screening. However, the incidence of Barrett's adenocarcinoma is expected to increase in the future and should be monitored closely.^10^ The prognosis of both gall bladder cancer and cholangiocarcinoma is poor. Even after complete resection, the 5‐year survival rate ranges from 8% to 40%.^14^ Early detection of underlying conditions such as biliary dilatation and stratification of high‐risk patients based on known risk factors such as obesity^14^ may be necessary to decrease the prevalence of gall bladder cancer and cholangiocarcinoma.

Excluding cancer, other chronic diseases account for a lower proportion of the disease burden; however, the prevalence of chronic diseases is expected to increase in the future. Regarding biliary diseases, obesity is related to cholesterol gallstones and is associated with an increased risk for gallbladder cancer. The prevalence of IBD^25^ and GERD^9^ is increasing in Japan, as they are in Europe and the United States.^25^

Our study provides comprehensive descriptive epidemiology of gastrointestinal diseases and predicts future situations. The limitations of our study are as follows. First, regarding the GBD data, future GBD studies could be improved by including detailed data on many factors such as the *H. pylori* eradication rates and pathological types of cancer. Second, there is a time lag of a few years in GBD data availability compared with vital registration systems. Lastly, we estimated 22 gastrointestinal chronic diseases using the data of the GBD study, but we did not include several acute conditions such as lower gastrointestinal bleeding and diverticulitis. Nonetheless, the GBD study involves data‐seeking efforts and data corrections, and its approach can enable comparisons of the tendency of disease burden among countries.

Our study provides comprehensive estimates of the burden of gastrointestinal diseases in a super‐aging society focusing on cancers and other chronic gastrointestinal diseases. Currently, colorectal, gastric, pancreatic, and liver cancers are the focus of early detection and treatment. The results provide comparable estimates that may help establish protocols to control gastrointestinal diseases in Japan.

## Ethics statement

Institutional Review Board approval was not required because this was a secondary analysis of publicly available GBD data.

## Supporting information


**Figure S1.** Crude death rates (per 10 000) for men combined in 1990, 2019, and 2035.

**Figure S2.** Crude death rates (per 10 000) for women combined in 1990, 2019, and 2035.

## Data Availability

The GBD data are publicly available.
