# Supplementary figures and images for: The burden of gastrointestinal diseases in Japan, 1990–2019, and projections for 2035
Source: JGH Open. 2023 Mar 7;7(3):221–7. doi: 10.1002/jgh3.12883 (PMC10037033; doi:10.1002/jgh3.12883)

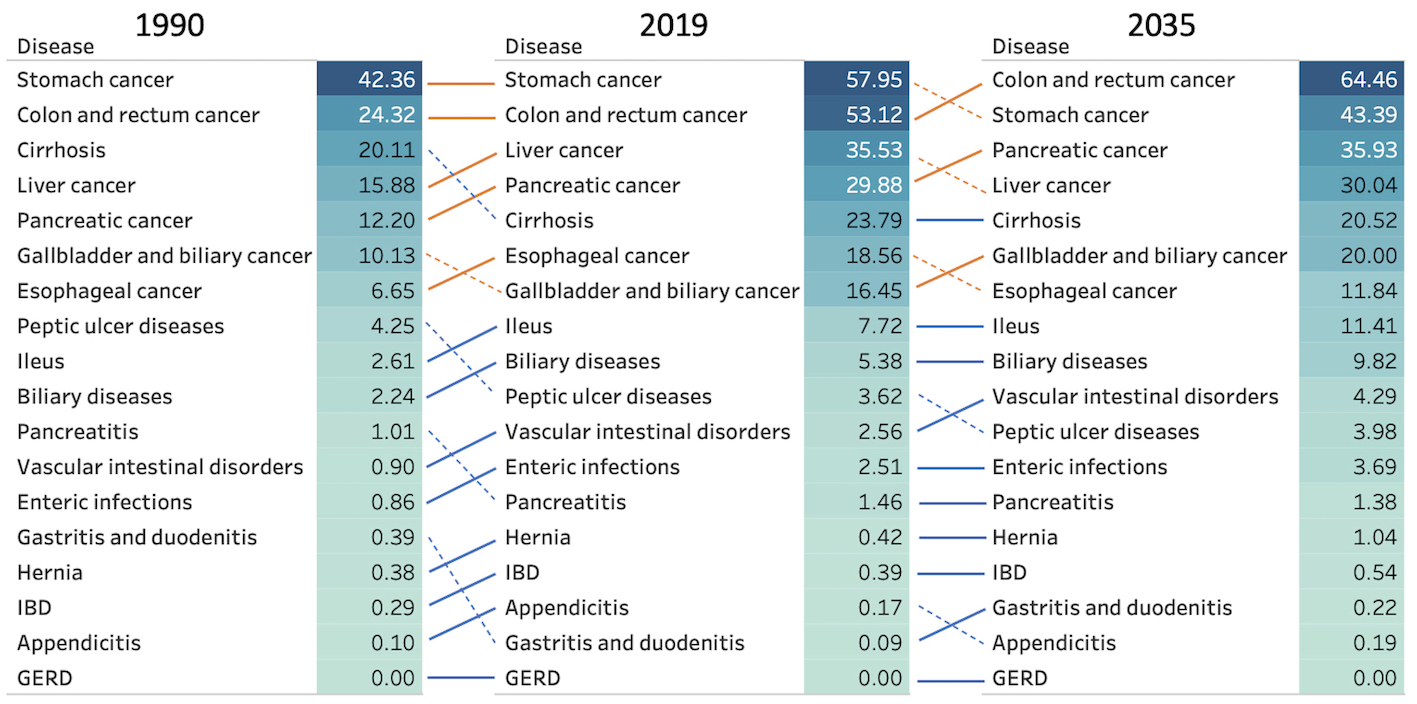

Supplement: Supplementary file 1 — Figure S1. Crude death rates (per 10 000) for men combined in 1990, 2019, and 2035. [file JGH3-7-221-s002.jpg]

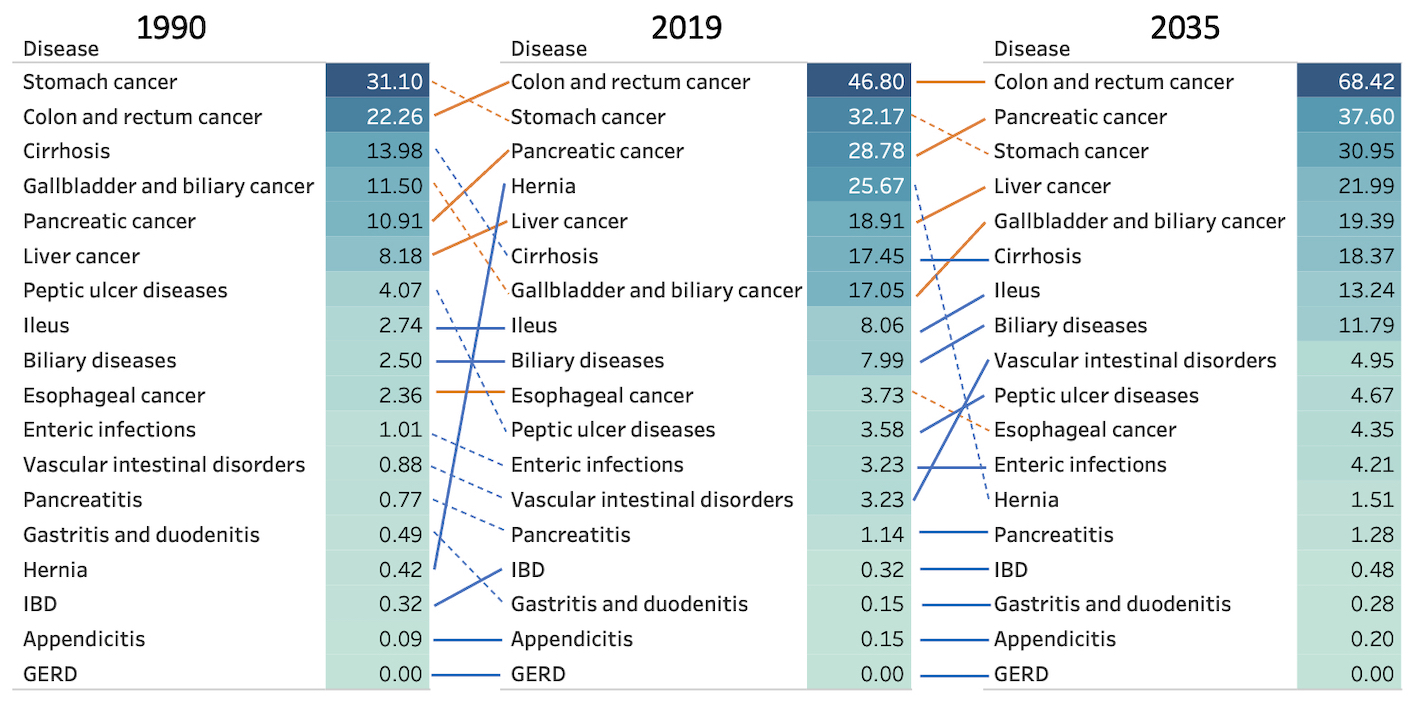

Supplement: Supplementary file 2 — Figure S2. Crude death rates (per 10 000) for women combined in 1990, 2019, and 2035. [file JGH3-7-221-s001.jpg]
